# Supreme laryngeal mask airway for cesarean section under general anesthesia: a 10-year retrospective cohort study

**DOI:** 10.3389/fmed.2023.1181503

**Published:** 2023-07-20

**Authors:** Zhiyu Geng, Chunqing Li, Hao Kong, Linlin Song

**Affiliations:** Department of Anesthesiology, Peking University First Hospital, Beijing, China

**Keywords:** anesthesia, obstetric, cesarean section, laryngeal mask, maternal outcomes, neonatal outcome

## Abstract

**Background:**

Previous research showed the use of supraglottic airways in obstetric anesthesia. The relevant evidence of laryngeal mask airway (LMA) on maternal and neonatal outcomes is still limited. We aimed to assess the maternal and neonatal outcomes when the LMA Supreme was used for cesarean section under general anesthesia.

**Methods:**

We included all patients who underwent general anesthesia for cesarean section between January 2010 and December 2019. Propensity score matching was used to reduce potential bias from non-random selection of airway intervention. The primary outcome was adverse maternal and neonatal outcomes defined as maternal regurgitation, aspiration, hypoxemia, and low neonatal Apgar scores. Secondary outcomes included patient admission to the intensive care unit, neonate required tracheal intubation, external cardiac massage, and admission to the neonatal intensive care unit.

**Results:**

A total of 723 patients were included in the analysis; of whom, 221 received Supreme laryngeal mask airway (LMA group) and 502 were intubated with an endotracheal tube (ETT group). After propensity score matching, 189 patients remained in each group. No episode of regurgitation and aspiration occurred in both groups. There was no difference in the rates of Apgar score below 7 at 1 min (14.3% LMA group vs. 15.3% ETT group, OR 0.931, 95% CI 0.574 to 1.510, *P* = 0.772) and 5 min (3.7% vs. 4.2%, OR 0.875, 95% CI 0.324 to 2.365, *P* = 0.792). No difference was observed in the secondary outcomes between the two groups.

**Conclusion:**

The LMA Supreme was not associated with higher adverse maternal and neonatal outcomes when compared to an endotracheal tube for cesarean section under general anesthesia. It might be considered an alternative to tracheal intubation in obstetric practice.

## Introduction

The laryngeal mask airway (LMA) is a useful supraglottic airway device for most procedures. It is simple and atraumatic to insert, with minimal hemodynamic response and a lower risk of airway complications when compared with tracheal intubation. High insertion success on the first attempt and low complication rate make its use widespread in clinical practice (1, 2).

As one of the second-generation LMA, the LMA Supreme has a gastric drain tube and higher oropharyngeal leak pressure which provides a satisfactory airway for positive pressure ventilation (2, 3). As protection from aspiration is improved, it has been used as an acceptable alternative to endotracheal tubes even in patients with potential regurgitation, such as pregnancy, morbid obesity, or laparoscopic surgery (4–6). Furthermore, it has proven valuable as a rescue device in managing difficult airway situations. Many reports have demonstrated successful or even life-saving use of LMA for failed tracheal intubation occurring in elective and emergent obstetric situations (7–10). Thus, the second-generation LMA is recommended for managing difficult and failed tracheal intubation in obstetric failed intubation guidelines (11).

The main issue with the LMA in obstetric anesthetic practice is the potential risk of regurgitation and aspiration, especially in the event of emergency situations. Although previous studies have reported the successful use of the LMA in cesarean section, the role of LMA in obstetric general anesthesia is, to date, still highly debatable (12–17).

Research on obstetric anesthesia should focus on reducing anesthesia-related morbidity and ensuring the safety of both the mother and neonate. Therefore, this cohort study aimed to assess maternal and neonatal outcomes when the LMA Supreme was used for cesarean section under general anesthesia. We hypothesized that the LMA Supreme is not associated with higher adverse maternal and neonatal outcomes when compared to the tracheal tube.

## Materials and methods

### Study design

Following ethical approval by the Ethics Committee of Peking University First Hospital (No. 2021-226, date of approval: 23 June 2021, Chairman: Professor Yanyan Yu), a retrospective cohort study was carried out on patients who received general anesthesia for cesarean section over a period of 10 years (January 2010 to December 2019). The requirement for writing informed consent was waived by the Ethics Committee. The manuscript is reported according to the Strengthening the Reporting of Observational Studies in Epidemiology (STROBE) statement (18).

### Study population

Peking University First Hospital is a tertiary university teaching hospital, and the patient volume is approximately 5,000–7,000 deliveries per year. We included all patients who received general anesthesia for cesarean section within the defined study period. Patients who met any of the following criteria were excluded: (1) gestational age < 28 weeks, (2) cesarean section for stillbirth, (3) insufficient neuraxial anesthesia rescued with intravenous sedation without airway intervention, and (4) missing data on the airway device employed.

In our clinical practice, combined spinal epidural anesthesia is the preferred method for cesarean section, and epidural labor analgesia is provided around the clock for 20 years. General anesthesia for cesarean section is mainly indicated for anesthetic factors (contraindications to neuraxial anesthesia and failed neuraxial block), obstetric factors (placental abruption/previa/accreta, fetal distress, and cord prolapse), and maternal factors (severe comorbidities). At the time of an emergency cesarean section, a neonatologist attends the operating room to provide neonatal evaluation and resuscitation when necessary.

The standard general anesthetic used for rapid sequence induction was propofol. Muscle relaxation was accomplished with succinylcholine, rocuronium, or cisatracurium, at the discretion of the attending anesthetist. Maintenance of anesthesia was achieved with propofol, nitrous oxide, and sevoflurane. Remifentanil or sufentanil is provided after clamping the umbilical cord and delivery of the baby. Antacid prophylaxis medication was not routinely used. Non-invasive blood pressure, electrocardiogram, pulse oximetry, capnography, and bispectral index were routinely monitored. These variables were recorded in our electronic anesthetic database at 10-s intervals.

### Data collection

Data were retrieved from the hospital's electronic medical database and the anesthetic information management system. The following data were collected: (1) demographic data included maternal age, height, weight, body mass index (BMI), gravidity and parity, gestational age, single/multiple gestations, American Society of Anesthesiologists (ASA) physical status, *in vitro* fertilization and embryo transfer (IVF-ET), history of pre-existing or pregnancy-associated disorders, and previous cesarean section. (2) Perioperative data included preoperative Mallampati classification, indications for general anesthesia, planned/urgent surgery, fast time, airway management technique, intraoperative pulse oxygen saturation (SpO_2_), neonatal delivery time, and complications related to airway management. (3) Maternal outcome data included regurgitation, aspiration, hypoxemia, composite morbidity, mortality, and intensive care unit (ICU) admission. (4) Neonatal outcome data included 1-min and 5-min Apgar scores, fetal birth weight, need for tracheal intubation or external cardiac massage, and admission to the neonatal intensive care unit (NICU).

### Exposure variable

Patients were allocated into two groups according to the airway devices used for general anesthesia: the LMA Supreme group (LMA group) and the endotracheal tube group (ETT group). In the LMA group, a gastric drainage tube was inserted *via* the gastric drainage aperture. After successful placement, the gastric drain tube was easily advanced, and gastric content was continuously suctioned during the surgery. In the ETT group, an ID 7 tracheal tube was intubated by direct laryngoscopy or video-laryngoscopy (video laryngoscopy has been routinely used in our institution since 2017). An effective airway was confirmed by auscultation and the presence of a square wave capnograph trace. If patients were converted from the tracheal tube to the LMA during the case, they were considered the LMA group.

### Outcome assessment

The primary outcome was adverse maternal and neonatal outcomes defined as maternal regurgitation, aspiration, hypoxemia, and neonatal Apgar score below 7 at 1 min and 5 min. The secondary outcomes included the following: (1) difficult airway and failed intubation; (2) neonate need for intubation, external cardiac massage, and admission to the neonatal intensive care unit (NICU); and (3) maternal admission to the intensive care unit (ICU).

Regurgitation was defined if clear or bile-stained fluid was seen in the mouth during the procedure or at the removal of the airway device. Aspiration was defined as either bile-stained fluid or particulate matter seen in the tracheobronchial tree with a fiberoptic endoscope or a postoperative chest radiological evidence was presented. Hypoxemia was defined as low pulse oxygen saturation (SpO_2_) ≤ 90% for at least 10 consecutive seconds. Difficult intubation was defined as requiring more than two attempts at intubation or documented as such, based on the opinion of the anesthetist. Failed intubation was defined as the inability to place a tracheal tube after multiple attempts with direct laryngoscopy or an alternative airway device.

Criteria for NICU admission were defined as any neonate with a 1-min Apgar score lower than 7, delivered by a high-risk parturient, low birthweight, and preterm delivery.

### Statistical analysis

Data were analyzed using SPSS 22.0 software (SPSS, Inc., Chicago, Illinois, USA). The Shapiro–Wilk test was used to test the hypothesis of normal distribution. Normally distributed continuous variables were described as the mean ± standard deviation and analyzed with a two-sided independent *t-*test. Non-normally distributed variables were described as medians (IQR) and analyzed using the Mann–Whitney *U* test. Categorical variables were described as numbers (proportion) and compared using Pearson's chi-square test or Fisher's exact test as appropriate.

We used propensity score-matched analysis to reduce confounding bias and potential baseline differences between the two groups. Only parturients with singleton pregnancies were included for propensity analysis. The propensity score matching process was carried out using a multivariable logistic regression model with the airway device as the dependent variable. The independent variables included the following potential confounders: age, BMI, ASA physical status, gestational age, preterm delivery, emergent surgery, fast time, maternal comorbidities, pregnancy-related conditions (hypertension and diabetes mellitus), and Mallampati score.

The LMA group patients were matched to the ETT group patients in a 1:1 ratio by applying the nearest-neighbor matching method without replacement, with a caliper width equal to 0.1 of the standard deviation of the logit of the propensity score. Absolute standardized differences (ASDs) were computed to examine the balance in covariates between the two groups. An ASD ≥0.158 (i.e., $1.96\times \sqrt{\left(n1+n2\right)/\left(n1\times n2\right)}$) was considered imbalanced between the two groups. The algorithm was able to match 189 patients in each group. After testing the sample for balance on variables in the model, a single variable logistic regression model was created to test the association of airway tools and maternal and neonatal outcomes. Odds ratios (ORs) and 95% CI for the main outcomes were reported. A two-sided *p* < 0.05 was considered statistically significant.

## Results

**Figure 1:**
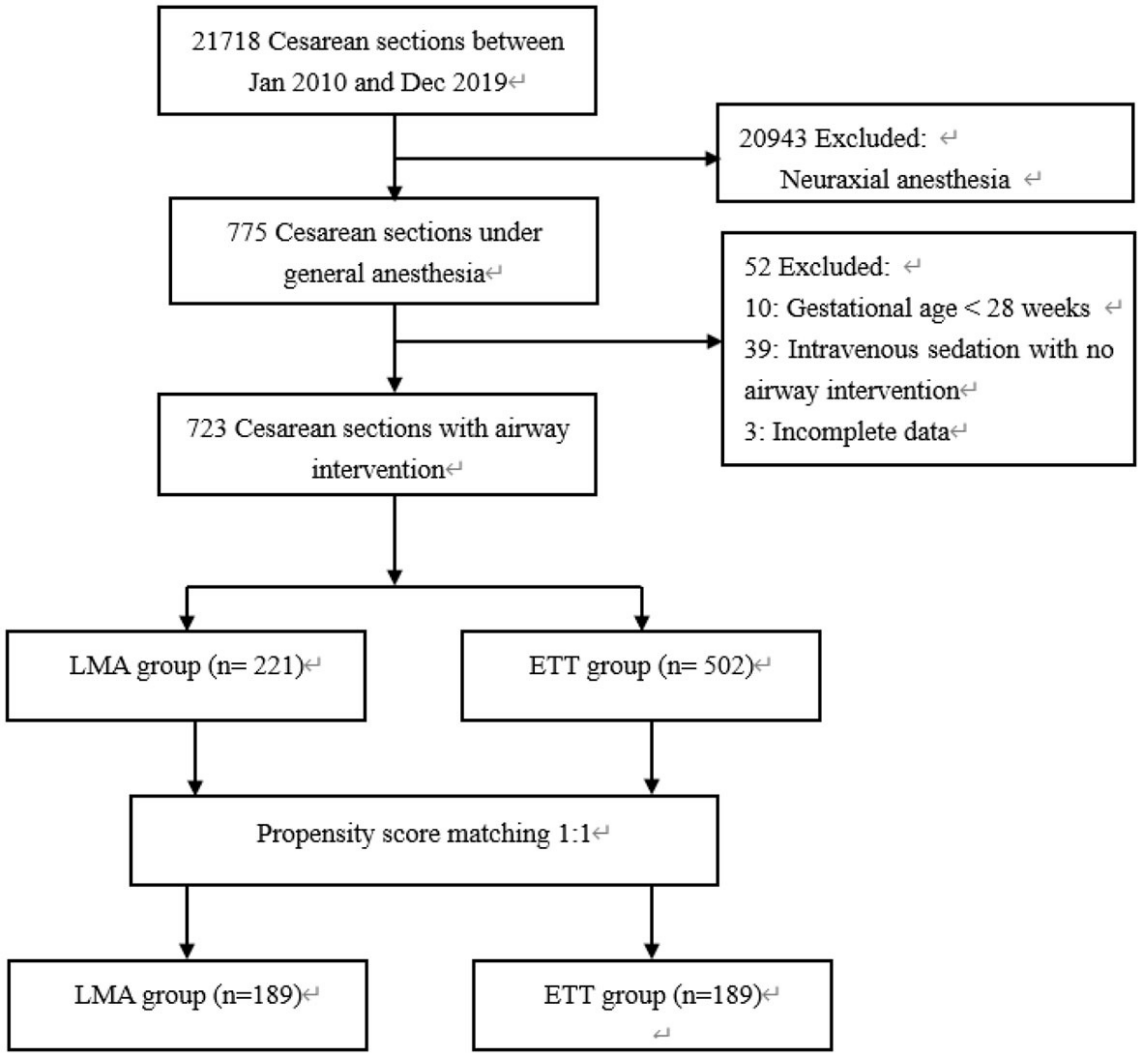
Participant flowchart.

**Figure 2:**
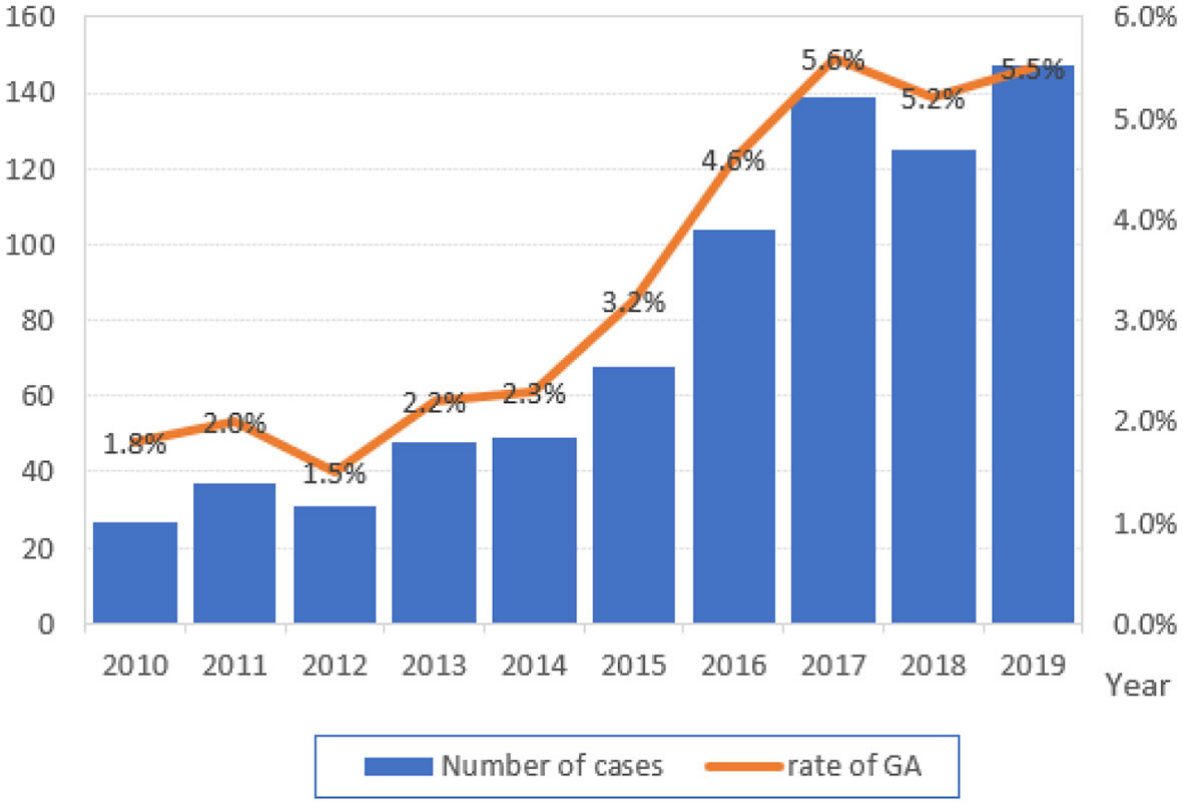
Total number and the annual rate of general anesthesia (GA) for cesarean section from 2010 to 2019.

The flow chart for the inclusion of patients is shown in Figure 1. A total of 21,718 cesarean sections were performed in the 10-year study period. The rate of general anesthesia was 3.6% (775 cases). We observed a trend of gradual increase in general anesthesia use, ranging from 1.8% in 2010 to 5.5% in 2019 over the past 10 years, and emergency surgery accounted for 57.8% (Figure 2). After exclusion, the study cohort included 723 cases that received airway intervention, of which 418 (57.8%) was emergency operation and 135 (18.7%) was category 1 cesarean section. When stratified by airway device, 221 (30.6%) patients received the LMA Supreme, and 502 (69.4%) patients were intubated with endotracheal tubes.

### Baseline characteristics

The clinical characteristics of the LMA group and the ETT group are presented in Table 1. There were significant differences between the two groups for the proportion of ASA physical status ≥ 3, preterm delivery, coexisting gestational diabetes mellitus, unfasted patient, and emergency cesarean section.

**Table 1:** Baseline characteristics of all patients.

**Variable**	**LMA group (*n =* 221)**	**ETT group (*n =* 502)**	**Z or *χ^2^***	***P* value**
Age (years)	32.0 (29.5–36.0)	33.0 (29.0–36.0)	−0.767	0.443
Height (cm)	162 (158–166)	163 (159–166)	−0.470	0.639
Weight (kg)	71.5 (66.0–81.0)	71.0 (66.0–81.0)	−0.537	0.591
BMI (kg/m^2^)	27.5 (24.9–30.9)	27.3 (25.3–30.5)	−0.313	0.754
ASA physical status ≥3	37 (16.7%)	146 (29.1%)	12.363	< 0.001
Gestation age (weeks)	38.3 (36.3–39.1)	38.0 (35.0–39.3)	−1.345	0.178
Preterm delivery	62 (28.1%)	191 (38.0%)	6.737	0.009
Maternal coexisting disease	89 (40.3%)	205 (40.8%)	0.02	0.887
Gestational hypertension	28 (12.7%)	80 (15.9%)	1.289	0.256
Gestational diabetes	65 (29.4%)	109 (21.7%)	4.977	0.026
Previous cesarean section	69 (31.2%)	124 (24.7%)	3.334	0.068
IVF-ET	20 (9.0%)	35 (7.0%)	0.942	0.332
Obese	63 (28.1%)	141(28.5%)	0.013	0.908
Unfasted	25 (11.3%)	128 (25.5%)	18.509	< 0.001
Emergency cesarean section	100 (45.2%)	318 (63.3%)	20.607	< 0.001
Category 1 cesarean section	27 (12.2%)	108 (21.5%)	8.733	0.003
Mallampati score			3.114	0.211
1	96 (43.4%)	187 (37.3%)		
2	113 (51.1%)	276 (55.6%)		
3	12 (5.4%)	39 (7.8%)		

Data are presented as median (IQR) and number (%) as appropriate.

ASA, American Society of Anesthesiologists; BMI, body mass index; IVF-ET, *in vitro* fertilization and embryo transfer; unfasted, defined as fast time < 6 h.

Of the cohort, no cases of regurgitation or aspiration of gastric contents were detected; four patients (0.55%) were identified as having difficult airways. One patient was intubated by a more experienced person using direct laryngoscopy on multiple attempts. Awake fiberoptic intubation was performed on another patient with an anticipated difficult airway. Failed intubation was encountered in two (0.3%) patients, both of whom were successfully rescued with a Supreme laryngeal mask airway. In total, 18 cases (2.5%) had hypoxemia, and 11 cases (1.5%) occurred before delivery. There was no anesthesia-related mortality during the study period.

### Propensity score matching

This cohort included 678 singleton pregnancies and 45 twin pregnancies. Only parturients with singleton pregnancies were included for propensity analysis. Thus, the final primary study cohort consisted of 678 cases. Before propensity score matching, standardized differences in baseline characteristics ranged between 0.035 and 0.509. After propensity score matching, all standardized differences were < 0.10, indicating that the groups were well matched (Table 2). The standardized differences between groups ranged from 0.000 to 0.086.

**Table 2:** Baseline characteristics of patients before and after propensity score-matching.

**Variable**	**LMA group (*n =* 200)**	**ETT group (*n =* 478)**	**ASD**	**LMA group (*n =* 189)**	**ETT group (*n =* 189)**	**ASD**
Age (years)	32.0 (29.3–36.0)	33.0 (29.0–36.0)	0.110	32.0 (29.0–36.0)	33.0 (30.0–36.0)	0.016
Height (cm)	162.5 (158.0–165.8)	162.5 (159.0–166.0)	0.035	163.0 (159.0–166.0)	163.0 (158.0–166.0)	0.012
Weight (kg)	71.0 (66.0–80.8)	72.0 (66.0–80.1)	0.075	71.0 (66.0–81.0)	72.0 (66.5–80.0)	0.053
BMI (kg/m^2^)	27.3 (24.8–30.8)	27.3 (25.2–30.5)	0.074	27.3 (24.8–30.8)	27.3 (25.6–30.4)	0.034
ASA physical status ≥3	35 (17.5%)	139 (29.1%)	0.304	35 (17.5%)	33(17.2%)	0.028
Gestation age (weeks)	38.5 (37.0–39.3)	38.1(35.0–39.3)	0.357	38.3 (37.0–39.3)	38.6 (37.1–39.4)	0.038
Preterm delivery	47 (23.5%)	175 (36.6%)	0.308	47 (24.9%)	42 (22.2%)	0.062
Maternal coexisting disease	81(40.5%)	193(40.4%)	0.003	74(39.2%)	80(42.3%)	0.065
Gestational hypertension	24 (12.0%)	74 (15.5%)	0.107	21 (11.1%)	25 (13.2%)	0.065
Gestational diabetes	61 (30.5%)	101 (21.1%)	0.203	52 (27.5%)	52 (27.5%)	0.000
Previous cesarean section	68 (34.0%)	131 (27.4%)	0.139	60 (31.7%)	57 (30.2%)	0.033
IVF-ET	13 (6.5%)	25 (5.2%)	0.051	13 (6.9%)	13 (6.9%)	0.000
Unfasted	21 (10.5%)	125 (26.2%)	0.509	21 (11.1%)	26 (13.8%)	0.086
Emergency cesarean section	89 (44.5%)	305 (63.8%)	0.388	88 (46.3%)	89 (47.1%)	0.011
Mallampati score			0.142			0.057
1	86 (43.0%)	176 (36.8%)		80 (42.3%)	82 (43.4%)	
2	104 (52.0%)	267 (55.9%)		99 (52.4%)	95 (50.3%)	
3	10 (5.0%)	35 (7.3%)		10 (5.3%)	12 (6.3%)	

Data are presented as median (IQR) and number (%) as appropriate.

ASA, American Society of Anesthesiologists; BMI, body mass index; IVF-ET, *in vitro* fertilization and embryo transfer; ASD, absolute standardized difference.

### Outcomes

Within the matched cohort, there were no episodes of regurgitation or aspiration. There were no differences in maternal hypoxemia (1.1% in the LMA group and 3.2% in the ETT group, OR 0.333, 95% CI 0.068 to 1.631, *P* = 0.153), Apgar scores below 7 at 1 min (14.3% vs. 15.3% in the LMA and ETT groups, respectively; OR 0.931, 95% CI 0.574 to 1.510, *P* = 0.772) and 5 min (3.7% vs. 4.2% in the LMA and ETT groups, respectively; OR 0.875, 95% CI 0.324 to 2.365, *P* = 0.792). In addition, 2.1% and 3.2% of neonates had 1-min Apgar scores below 3 in the LMA and ETT groups, respectively (OR 0.600, 95% CI 0.145 to 2.475, *P* = 0.721).

For the secondary outcomes, no significant differences were observed with regard to the rate of neonates admitted to the NICU (29.6% vs. 32.3%; *P* = 0.578), the need for endotracheal intubation (3.2% vs. 3.2%; *P* > 0.999), and maternal admission to the ICU (5.3% vs. 6.4%; *P* = 0.644) between the two groups (Table 3).

**Table 3:** Neonatal and maternal outcomes data after propensity score-matching.

	**LMA group (*n* = 189)**	**ETT group (*n* = 189)**	***P* value**	**OR (95% CI)**
Apgar score				
1-min ≤ 7	27 (14.3%)	29 (15.3%)	0.772	0.931 (0.574–1.510)
1-min ≤ 3	3 (1.6)	5 (3.2)	0.721	0.600 (0.145–2.475)
5-min ≤ 7	7 (3.7%)	8 (4.2%)	0.792	0.875 (0.324–2.365)
Regurgitation	0	0		
Aspiration	0	0		
Neonatal need intubation	6 (3.2%)	6 (3.2%)	1.000	>0.999 (0.328–3.045)
Neonatal need cardiac massage	1 (0.5%)	0	0.317	
Neonatal NICU	56 (29.6%)	64 (32.3%)	0.578	0.918 (0.679–1.241)
Neonatal NICU due to low Apgar score	25 (13.2%)	25 (13.2%)	1.000	>0.999 (0.597–1.676)
Neonatal weight (g)	3,240 (2,720–3,545)	3,190 (2,770–3,500)	0.612	
Difficult intubation	1 (0.5%)	1 (0.5%)	1.000	>0.999 (0.063–15.871)
Maternal hypoxemia before delivery	2 (1.1%)	6 (3.2%)	0.153	0.333 (0.068–1.631)
Maternal ICU	10 (5.3%)	11 (6.4%)	0.644	0.823 (0.279–1.594)
Maternal morbidity	1 (0.5%)	2 (1.1%)	0.562	0.500 (0.046–5.468)
Maternal mortality	0	0		

Data are expressed as number (%) or median (IQR).

NICU, neonatal intensive care unit; ICU, intensive care unit.

## Discussion

Our study showed that compared with tracheal intubation, the LMA Supreme was not associated with higher adverse maternal and neonatal outcomes for cesarean section performed under general anesthesia. No regurgitation or aspiration occurred in both groups, and the rates of neonatal Apgar scores below 7 at 1 min and 5 min were similar between the two groups. In addition, the incidences of maternal hypoxemia, admission to the ICU, neonates admitted to the NICU, and neonates requiring tracheal intubation did not differ significantly between the two groups.

Due to its rapid and predictable onset, general anesthesia is commonly used especially in urgent cesarean section. In the circumstance of a category 1 cesarean section for fetal distress, general anesthesia is associated with the most rapid operating room-to-incision interval when compared to spinal anesthesia and epidural top-up with a functioning catheter in place (19, 20). In our cohort, the rate of general anesthesia for cesarean section escalated from 1.8% to 5.5% over the past 10 years. Among these, emergency and category 1 cesarean sections accounted for 57.8% and 18.7%, respectively.

Previous clinical research showed successful use of the different types of LMA for selected low-risk patients and urgent cesarean sections (12–15). LMA could rapidly establish an effective airway, and its high rate of success at the first attempt is highly desirable. No aspiration, bronchospasms, or hypoxemic episodes were observed. Only one case of regurgitation after insertion of the ProSeal LMA was detected in more than 6,000 cases. Although rapid sequence induction and tracheal intubation are considered the gold standard of general anesthesia for pregnant patients, early insertion of a supraglottic airway device (preferably a second-generation LMA with better protection against aspiration) as a rescue airway device is recommended in obstetric failed tracheal intubation guidelines (11). In our real-world study, the LMA Supreme could establish a reliable airway even in some high-risk obese patients with a BMI >30 kg/m^2^ or an unpredicted difficult airway. We did not detect any clinical evidence of regurgitation or aspiration, probably due to more experienced anesthetists who were familiar with this airway device in our unit. The LMA Supreme was routinely used in our gynecologic laparoscopic surgery in our daily practice. With more training opportunities, our practitioners had a good experience in handling the LMA Supreme.

Studies comparing the LMA Supreme with the tracheal tube on maternal and neonatal outcomes are limited. In a recent retrospective observational study, the author evaluated the use of the LMA Supreme for emergency cesarean section and reported that 37 of 1,137 (3.25%) neonates were intubated (10). As a prospective randomized controlled trial comparing the LMA Supreme with tracheal tubes on maternal and neonatal outcomes for a cesarean section may be ethically impractical to perform, we used propensity score-matched analysis to reduce potential confounding bias. Our results demonstrated that the LMA Supreme could be used safely and effectively in cesarean section and no adverse maternal and neonatal outcomes increased when compared with conventional tracheal intubation.

Transplacental transfer of anesthetic drugs, maternal hypoxia resulting from difficult mask ventilation and failed intubation, and maternal neuroendocrine stress response to tracheal intubation are potential reasons for neonatal depression during cesarean section with general anesthesia. Factors that affect fetal exposure to maternal drugs are the time between induction of anesthesia and clamping of the umbilical cord and uterine incision to delivery time. A prolonged uterine incision to delivery time is associated with an increase in the incidence of fetal acidosis due to uteroplacental vasoconstriction (21). In urgent cases, the maximum decision-to-delivery interval of 30 min is recommended to improve early neonatal outcomes (22). The LMA Supreme has a potential advantage over the tracheal tube in terms of rapid airway establishment, hemodynamic stability, and smooth emergence profile that could confer further benefit in obstetric situations (23–25). More rapid insertion, fewer anesthetic drugs used for stable hemodynamics, and fewer airway complications are of critical importance for high-risk parturients, such as concurrent cardiovascular disease, morbid obesity, and preeclampsia (26–29).

For obstetric airway, we still emphasize that avoiding oxygen desaturation and regurgitation is of the utmost importance regardless of which airway device is used. The 2015 OAA/DAS obstetric airway guideline recommended head-up position, preoxygenation to achieve end-tidal oxygen concentrations of ≥ 90%, and gentle bag/facemask ventilation (maximal inflation pressure < 20 cmH_2_O) during rapid sequence induction (11). Recent literature has suggested that high-flow nasal oxygen therapy (HFNO) provides longer safe apnea times, higher PaO_2_, and end-tidal oxygen concentration after intubation in parturients and thus could be considered a safe method of oxygenation during rapid sequence induction for parturients undergoing general anesthesia (30, 31).

As we know, pregnant patients are considered “full stomach” due to pregnancy-related physiological changes. Before the routine use of ultrasound as a screening tool to accurately assess gastric content in obstetric patients, whether the second-generation LMA could replace tracheal intubation as a primary airway device remains a more controversial topic (16, 17, 32). Although high-quality research supporting recommended use of the LMA Supreme is still lacking, our obstetric anesthetist should be encouraged to use this airway instrument in obstetric clinic practice. The correct position of the LMA Supreme is vital to achieving a good seal to prevent fluid in the hypopharynx from entering the airway. Malposition can lead to gastric insufflation and block pharyngeal drainage if regurgitation occurs. Inserting a gastric tube through a separate drainage tube can be used as a valid position test, and continuous suction could further reduce gastric content and intragastric pressure, thus decreasing the intraoperative risk of reflux and aspiration (2). In addition to a theoretical prerequisite, good experience in airway devices is of particular importance in potentially challenging situations.

The study has some limitations. First, as a retrospective analysis, we did not record the oropharyngeal leak pressure, time to effective ventilation, and number of attempts, hence, we were unable to evaluate the total success rate of insertion. Second, in our cohort, women were generally of small stature and fasted. Therefore, the applicability of these results to other patient populations is unknown. Finally, the study may be underpowered for a relatively small sample size. Further prospective large sample trials are still needed to provide sufficient evidence to recommend the use of the LMA Supreme for cesarean section.

## Conclusion

Our results demonstrated that the LMA Supreme was not associated with higher adverse maternal and neonatal outcomes when compared to endotracheal tubes for cesarean section under general anesthesia. It might be considered an alternative to tracheal intubation in obstetric practice.

## Data availability statement

The original contributions presented in the study are included in the article/supplementary material, further inquiries can be directed to the corresponding author.

## Ethics statement

The studies involving human participants were reviewed and approved by the Ethics Committee of Peking University First Hospital. The Ethics Committee waived the requirement of written informed consent for participation.

## Author contributions

ZG: contribution to conception and design of the study. CL and HK: analysis and interpretation of data. ZG and LS: writing manuscript. All authors contributed to the article and approved the manuscript.
